# Type-1 Grade 2 Multi-Focal Gastric Neuroendocrine Tumors Secondary to Chronic Autoimmune Gastritis

**DOI:** 10.3389/fmed.2022.856125

**Published:** 2022-06-17

**Authors:** Ziqi Yu, Aiyao Wang, Chong Hu, Tao Yu, Jianyong Chen

**Affiliations:** ^1^Munich Medical Research School, Ludwig Maximilian University (LMU) of Munich, Munich, Germany; ^2^Department of Gastroenterology and Hepatology, The First Affiliated Hospital of Nanchang Medical College, Jiangxi Provincial People’s Hospital, Nanchang, China

**Keywords:** chronic autoimmune gastritis, atrophic gastritis, gastric neuroendocrine tumors, type-1 gastric carcinoid, G cell hyperplasia, ECL cell

## Abstract

**Background:**

Chronic autoimmune gastritis (CAG) refers to chronic atrophic gastritis due to autoimmunity. Loss of gastric glands in CAG results in hypergastrinemia and achlorhydria leading to Vitamin B_12_ deficiency and hyperplasia of G cells and enterochromaffin-like (ECL) cells. Vitamin B_12_ deficiency could cause pernicious anemia and subacute combined degeneration, while G cells and ECL cells hyperplasia might develop gastric neuroendocrine tumor (G-NET).

**Case Presentation:**

A 35-year-old Chinese female presented with multi-focal type-1 Grade 2 (G2) NETs with a 14-year history of pernicious anemia and subacute combined degeneration.

**Conclusion:**

Here, we report a rare case of a Chinese patient presenting G-NET combined with pernicious anemia and subacute combined degeneration, which are secondary to chronic autoimmune gastritis. This case also illustrates the importance of routine gastroscopy in patients with Vitamin B_12_ deficiency.

## Background

Atrophic gastritis is defined as chronic gastric inflammation referring to the disappearance of the gastric glands regardless of the metaplasia (1, 2). It mainly consists of a rare form of autoimmune gastritis (gastritis A) and a common form of *Helicobacter pylori* (H. pylori) associated with gastritis (gastritis B) (2, 3). Normally, for chronic autoimmune gastritis (CAG), women would have a higher prevalence with a 3:1 ratio to males (2, 4). The research found that the histologic change in CAG is a risk factor for gastric neuroendocrine tumor (G-NET) development (5, 6), and in patients with CAG, the annual incidence of G-NET was 0.68% per person-year (7, 8).

The G-NETs are rare neoplasms with origin in the peripheral neuroendocrine system in the stomach (9). G-NET could be subdivided into 4 types. Type-1 G-NETs predominantly occurred in females, representing 70–80% of G-NETs, and are gastrin-dependent (10). Normally, type-1 G-NETs are small multiple tumors around 1–2 cm located in the gastric body or fundus (6, 9, 11). Type-2 G-NETs take up 5% of the G-NETs. They are also gastrin dependent and normally associated with Zollinger-Ellison syndrome (ZES) or multiple endocrine neoplasias 1 (MEN1). They are also small and multiple tumors but have an equal prevalence in males and females (6, 9–11). Type-3 G-NETs comprise 10–15% of the G-NETs and are more frequently found in males. They are sporadic and gastrin-independent. They are normally single and larger than 1 cm, with high metastasis potential. In total, 25–40% of Type-3 G-NETs are malignant (6, 9, 10). The newly defined and rarest Type-4 G-NETs are also sporadic and gastrin-independent but with the highest metastasis potential (6, 10).

Besides, according to the mitotic rate and Ki-67 index, the World Health Organization (WHO) classified the NETs into Grade 1 to Grade 3. NETs with a low mitotic index (< 2) and Ki-67 proliferation index (< 3%) are classified as NET G1, NETs with the intermediate mitotic rate (2–20) and Ki-67 index (3–20%) are defined as NET G2, and NETs with the high mitotic rate (> 20) and Ki-67 index (> 20%) are named as NETs G3 (12).

In this report, we described a rare case of multiple Type-1 G2 G-NETs secondary to CAG. This patient also presented long-term pernicious anemia and subacute combined degeneration.

## Case Presentation

**FIGURE 1:**
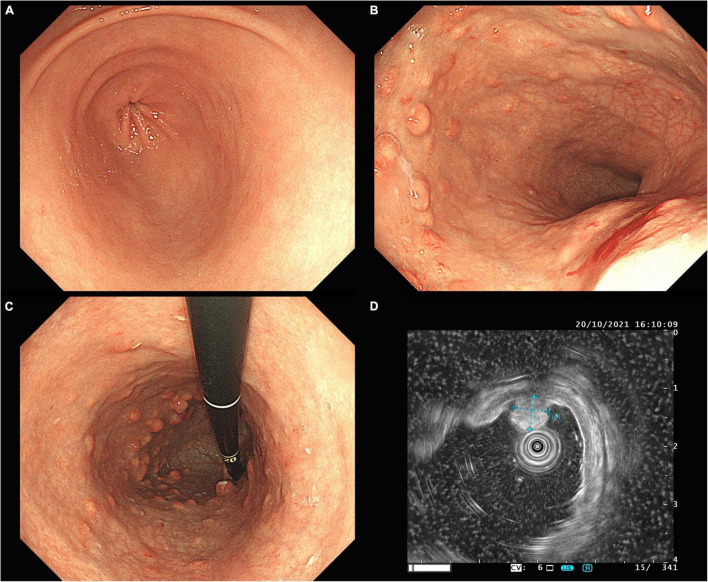
Routine gastroscopy revealed smooth mucosa in the gastric antrum **(A)**, absence of gastric fold along the greater curvature of the gastric body **(B)**, pale appearance of mucosa, increased visibility of vasculature, and multiple gastric submucosal bulges within gastric body, and fundus **(B,C)**, EUS confirmed hyperechoic NETs originated from the muscularis mucosa, with an intact muscularis propria **(D)**.

**FIGURE 2:**
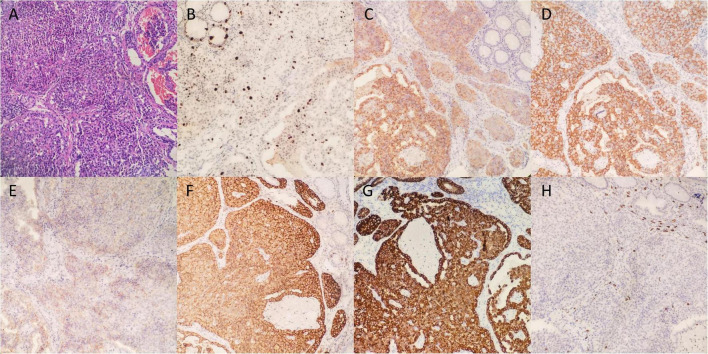
Histological findings of pathological biopsies. **(A)** Hematoxylin and eosin staining (200x). **(B)** Ki-67 staining revealed the Ki-67 index was 15% and mitotic rate was 12. The carcinoid showed positive staining of CgA **(C)**, Syn **(D)**, CD56 **(E)**, SSTR2 **(F)**, CK **(G)**, and negative staining for CD117 **(H)**.

A 35-year-old female with discomfort and fullness in the upper abdomen was admitted to our hospital. The patient was admitted due to fatigue in the lower limbs 14 years ago. She was diagnosed with pernicious anemia and subacute combined degeneration of the spinal cord in an outside hospital (laboratory results unavailable). Vitamin B_12_ and folic acid supplements were taken discontinuously but without symptomatic improvement. The patient has no family history of gastric disease. On admission, the patient was conscious and afebrile with normal vital signs. She has pale palpebral conjunctivas and fingernails with no other remarkable findings. Routine gastroscopy revealed autoimmune gastritis, multiple gastric submucosal bulges, and no endoscopic features related to H. pylori status according to the Kyoto classification (Figures 1A–C). Both the ^13^C-Urea breath test (UBT) and rapid urease test showed negative results. Laboratory tests showed moderate anemia with decreased hemoglobin (80 g/L), serum iron (4 μmol/L) and serum ferritin of 6.17 ng/ml; normal vitamin B_12_ (1,271.70 pg/ml) and folic acid (> 30 ng/ml) level and elevated gastrin 17 (> 60 pmol/L), decreased pepsinogen I (PG I) (12.01 μg/L), normal pepsinogen II (PG II) (4.11 μg/L), lower PG I/II ratio (2.92), negative PCA (anti-parietal cell antibody), and negative IFA (anti-intrinsic factor antibody). Liver and kidney tests, urinalysis, stool routine tests, and tumor markers tests (CEA, AFP, CA19-9, and CA125) were unremarkable. The electrocardiogram showed sinus rhythm and frequent premature ventricular contractions. Thyroid ultrasound revealed no abnormalities. Cranial CT scan (plain scan) and chest and abdominal CT scan (plain and contrast-enhanced scan) revealed no abnormalities, except mild splenomegaly. Endoscopic ultrasonography (EUS) revealed multiple submucosal bulges (Figure 1D), which were confirmed as G2 NETs in the gastric fundus, gastric body junction, and gastric body by histopathological examination of endoscopic biopsies (Figure 2). We recommended endoscopic surveillance, endoscopic submucosal dissection, or somatostatin analogs for the treatment of the patient. The patient and the family chose endoscopic surveillance for further treatment. Therefore, we gave the symptomatic treatment of vitamin B_12_, iron, and folic acid supplement and arranged a follow-up of gastroscopic surveillance every 6 months.

## Discussion and Conclusion

The CAG is a chronic inflammatory disease that is due to an autoimmune parietal cell antibody targeting H^+^, K^+^ -ATPase on parietal cell or intrinsic factor. The infiltration of immune cells results in the destruction of parietal cells and other structures on oxyntic mucosa, which further leads to the destruction of the gastric gland (3, 13, 14).

**FIGURE 3:**
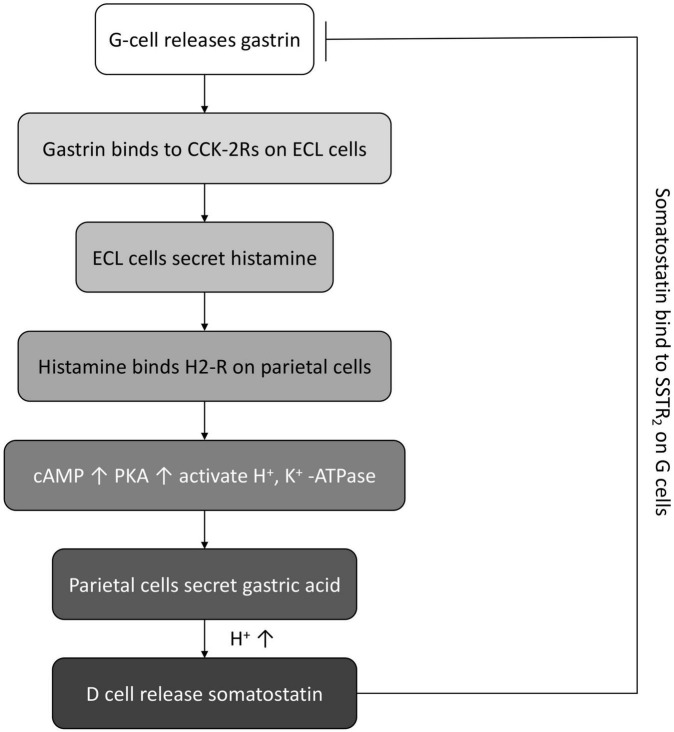
The schematic diagram of normal feedback loop between gastrin, gastric acid, and somatostatin.

In normal conditions, the antral G cells would secrete gastrin, which then binds to the cholecystokinin (CCK)-2 receptor on enterochromaffin-like (ECL) cells to stimulate the histamine release from ECL cells. Then, histamine binds to the G protein-coupled receptor H2 receptors on parietal cells. The activation of G protein-coupled receptor H2-R triggers the production of cAMP and the PKA signaling and further activates the H^+^, K^+^ -ATPase to pump out H^+^, recycle K^+^, and the concomitant secretion of Cl^–^, leading to the secretion of gastric acid. Along with the enhancement of acidity, the D cells are activated and release the inhibitory somatostatin, which in turn binds the somatostatin receptor type 2 (SSTR_2_) on G cells to inhibit the gastrin secretion (11, 15) (Figure 3).

However, regarding CAG, due to the destruction of parietal cells, the secretion of gastric acid and intrinsic factors are impaired. Gastric acid could exert multiple functions, such as Killing and preventing bacteria, digesting protein, and also inducing absorption of iron and vitamin B_12_ (15). The binding of intrinsic factors and vitamin B_12_ is also of vital importance to the absorption of vitamin B_12_ (16). Vitamin B_12_ is a co-factor in the reaction of the 5-methyl tetrahydrofolate (THF) converting to THF and also the conversion of homocysteine into methionine (17, 18). Deficiency in vitamin B_12_ could, on one hand, impair the *de novo* synthesis of purine and thymidylate, on the other hand, interfere with the DNA methyltransferase (19). Therefore, the deficiency of vitamin B_12_ could inhibit DNA synthesis and DNA repair after damage. Deficient DNA synthesis and repairment in pre-erythroblasts result in megaloblastic anemia; Vitamin B_12_ deficient megaloblastic anemia is also called pernicious anemia (20). Pernicious anemia could be asymptomatic for 4–5 years since the soluble vitamin B_12_ cobalamin could be reserved in the liver and could be used after vitamin B_12_ deficiency (18). Our patient has a 14-year-history of pernicious anemia, indicating a long-term history of CAG. Besides, vitamin B_12_ also plays a role in the synthesis of myelin sheaths that conducts nerve impulses (18). Therefore, vitamin B_12_ deficiency might also result in progressive fatigue, weakness, and numbness in the distal limbs. Unsteady gait, hearing loss, and urinary disorders might also be observed in these patients (18, 21). Our patient also reported a history of fatigue in lower limbs and subacute combined degeneration, this is also due to the neurological effect of vitamin B_12_ deficiency.

Moreover, deprived gastric acid secretion also results in the continuous secretion of gastrin and loss of negative feedback exerted by somatostatin by D cells. Since gastrin can stimulate cell proliferation, migration, and inhibit apoptosis (22, 23), continuous release of gastrin results in the hyperplasia of G cells and ECL cells. G cell and ECL cell hyperplasia could further induce the type-1 NETs (24). Type-1 NETs are mostly classified as Grade 1 (10).

The diagnosis criteria of CAG introduced in this study are mainly according to the Japanese criteria with at least two of the following three standards: (1) endoscopic reverse atrophy (severe atrophy in the corpus, while no or mild atrophy in the antral area); (2) hypergastrinemia, significantly decreased PG (pepsinogen) I or PG I/II, or positive PCA/IFA; (3) existence of ECL hyperplasia. Standard 1 is a must include standard (25). The laboratory result of our patient revealed elevated gastrin level, decreased PG I and decreased PG I/II level, and the gastroscopy exhibited reverse atrophy and multiple gastric submucosal bulges. Immunohistochemical expression showed a 15% Ki-67 index, a mitotic rate of 12, negative CD117 (−), positive epithelial marker CK (+), positive neuroendocrine markers synaptophysin (Syn) (+), positive CD56 (+), chromogranin A (CgA) (+), and positive prognosis marker SSTR2 (+) (Figure 2), indicating that our patient had multi-focal and well-differentiated epithelial Type-1 Grade2 G-NETs caused by CAG, which is a rare form in Type-1 G-NETs.

For treatment, simple endoscopic surveillance and endoscopic resection are recommended for type-1 G-NETs that are smaller than 20 mm by both National Comprehensive Cancer Network (NCCN) and European Neuroendocrine Tumor Society (ENETS) (26, 27). Antrectomy is also a potential treatment for recurrent or multi-focal G-NETs as it can eliminate the hypergastrinemia caused by G cells and also prevent the hyperplasia of ECL cells. However, the risks of complications should be made known to the patients (28). For well-localized G-NETs, endoscopic mucosal restriction (EMR) and endoscopic submucosal dissection (ESD) have been recommended. The complete resection rate of EMR and ESD was 69 and 86%, respectively (29). Somatostatin analogs, which provide negative feedback by inhibiting gastrin secretion from G cells are also effective in treating type-1 G-NETs. However, they are not recommended due to the recurrence after cessation of medication and the high costs (30). In this case, our patient has type-1 G2 multi-focal G-NETs, with a size under 20 mm. EUS results indicated that the lesion is limited within the mucosa; meanwhile, cranial, chest, and abdominal CT scans exhibited no distant metastasis. The immunohistochemical result also showed positive SSTR2. Therefore, we suggested endoscopic surveillance, EMR, or somatostatin analogs for the treatment. However, the patient rejected EMR or somatostatin analogs treatment. Routine endoscopy was arranged for surveillance.

In conclusion, we report a rare case of type-1 G2 multi-focal G-NETs combined with pernicious anemia due to CAG. According to the medical history, the patient might have CAG underdiagnosed for more than 14 years. This also results in the development of type-1 G-NETs. Therefore, it is also suggested that patients with unknown reasons for vitamin B_12_ deficiency could undergo gastroscopy to exclude autoimmune gastritis.

## Data Availability Statement

The original contributions presented in this study are included in this article/supplementary material, further inquiries can be directed to the corresponding author/s.

## Ethics Statement

The studies involving human participants were reviewed and approved by the Ethics committee of Jiangxi Provincial People’s Hospital. The patients/participants provided their written informed consent to participate in this study.

## Author Contributions

ZY reviewed the literature and wrote the manuscript. AW performed the clinical endoscopic test. CH and TY collected the patient information. JC gave suggestions for revision. All authors have read and approved the final manuscript.

## Conflict of Interest

The authors declare that the research was conducted in the absence of any commercial or financial relationships that could be construed as a potential conflict of interest.

## Publisher’s Note

All claims expressed in this article are solely those of the authors and do not necessarily represent those of their affiliated organizations, or those of the publisher, the editors and the reviewers. Any product that may be evaluated in this article, or claim that may be made by its manufacturer, is not guaranteed or endorsed by the publisher.
